# Association between visceral adiposity and *DDX11* as a predictor of aggressiveness of small clear-cell renal-cell carcinoma: a prospective clinical trial

**DOI:** 10.1186/s40170-021-00251-y

**Published:** 2021-04-06

**Authors:** Jee Soo Park, Won Sik Jang, Jongchan Kim, Seung Hwan Lee, Koon Ho Rha, Won Sik Ham

**Affiliations:** Department of Urology and Urological Science Institute, Yonsei University College of Medicine, Seoul, Republic of Korea

**Keywords:** Adipose tissue, Clear-cell renal-cell carcinoma, Intra-abdominal fat, Obesity, Small renal mass

## Abstract

**Background:**

Visceral fat produces several hormones and cytokines associated with carcinogenesis and tumor progression. Herein, we investigated the association between visceral adiposity and target-gene mRNA expression in patients with localized small clear-cell renal-cell carcinoma (ccRCC).

**Methods:**

We included 200 patients with localized clinical T1a stage ccRCC who had undergone nephrectomy from November 2018 to November 2020 in a prospective clinical trial (NCT03694912). Visceral, subcutaneous, and total adipose tissue in these patients was measured via preoperative computerized tomography of the mid-third lumbar vertebra region. We then examined the association between adiposity and the mRNA levels of *PBRM1*, *BAP1*, *SETD2*, *KDM5C*, *FOXC2*, *CLIP4, AQP1, DDX11, BAIAP2L1,* and *TMEM38B* in matched frozen tumor tissues and plasma samples.

**Results:**

Upon the stratification of patients into quartiles according to their relative visceral adiposity, high visceral adiposity was found to be significantly associated with low ISUP grade (*P* = 0.004). Multivariate logistic regression analysis revealed a significant association between frozen tissue *DDX11* expression and high visceral adiposity (OR 0.676, 95% CI 0.587–0.779, *P* < 0.001). Moreover, frozen tissue *DDX11* expression was significantly associated with high ISUP grade (OR 1.556, 95% CI 1.223–1.981, *P* < 0.001). The frozen tissue mRNA expression of *DDX11* was identified as a biomarker for visceral adiposity and cancer aggressiveness.

**Conclusions:**

The results obtained herein will aid in inferring the aggressiveness of small ccRCCs, represented by ISUP nuclear grade, in clinical practice. Our findings indicated that *DDX11* and visceral fat play active roles in small ccRCC. These roles should be examined in future studies for the possible use of *DDX11* and visceral fat as prognostic biomarkers in the treatment of patients with ccRCC.

**Trial registration:**

ClinicalTrials.gov, NCT03694912, Registered 3 October 2018.

**Supplementary Information:**

The online version contains supplementary material available at 10.1186/s40170-021-00251-y.

## Background

Renal-cell carcinoma (RCC) is the sixth and tenth most common type of cancer in men and women worldwide, respectively [1]. Clear-cell RCC (ccRCC) represents the most prevalent type of RCC, and it is characterized by mutations in genes governing the hypoxia signaling pathway. Several studies have identified specific genetic variances, and possible biomarkers for ccRCC, leading to therapeutic innovations [2]. However, the tumor biology of most commonly identified masses, which are ≤ 4 cm in diameter and classified as small renal masses (SRMs), is poorly understood [3, 4]. A wide variety of therapeutic modalities is used against SRMs, including surgical tumor resection, ablation, as well as active surveillance in some instances. Therefore, understanding the molecular characteristics and pathogenesis of SRMs is important for identifying specific biomarkers and selecting the optimal therapeutic options in clinical settings [4].

Although obesity represents a well-established risk factor regarding RCC [5, 6], the detailed association between obesity and the prognosis of RCC patients is still controversial and unclear. Some studies have reported improved prognoses in overweight patients [6–12], whereas other studies have reported no association between the clinical course of RCC and body weight [13, 14]. Regarding SRMs, the prognosis of RCC is inversely proportional to the body mass index (BMI), indicating that the tumor biology of SRMs is distinct from that of non-SRMs.

Previous studies have mostly employed BMI as a measure of obesity; however, BMI cannot be used to distinguish between fat, muscle, and bone. Furthermore, BMI does not provide any information on fat distribution. The measurement of visceral fat can be used to assess true obesity because visceral fat is the largest endocrine organ in the body, producing several hormones and cytokines related to carcinogenesis and tumor progression [15].

Previously, we found that several potential prognostic biomarkers are considerably up- or downregulated in ccRCC, and can be used to identify the aggressive clinical T1-stage [16, 17]. Total RNA sequencing data of 24 ccRCC patients (12 patients each with and without aggressive characteristics) revealed 10 genes highly upregulated or downregulated associated with aggressive disease. Among these 10 genes, DDX11 was significantly upregulated in aggressive ccRCC and was associated with low cancer-specific survival and high recurrence rate [17]. In this study, we aimed to determine the association between visceral adiposity and the mRNA expression of potential biomarkers using frozen tumor tissues and preoperative plasma of patients with small ccRCC. The present study presents information regarding the development of rapid and straightforward techniques for the evaluation of the aggressiveness of small ccRCCs in clinical practice.

## Materials and methods

### Patients and tissues

All procedures involving human participants were performed in accordance with the ethical standards of the institutional and/or national research committee as well as the 1964 Helsinki Declaration and its later amendments or comparable ethical standards. The study was approved by the Institutional Review Board of the Yonsei University Health System (project no: 4-2018-0753). Written informed consent was obtained from all patients, and the manuscript does not contain any person’s data in any form.

The present study included 200 patients with small localized ccRCC (≤ 4 cm, pT1aN0M0), who were treated via nephrectomy only, and for whom frozen tumor tissues and matching preoperative plasma samples were available from a prospective study (ClinicalTrials.gov Identifier: NCT03694912) conducted between November 2018 and November 2020.

The inclusion criteria used were as follows: (1) localized small ccRCC (≤ 4 cm, pT1aN0M0); (2) availability of information on preoperative height and weight; (3) availability of preoperative computed tomography (CT) data and follow-up for more than 1 year; and (4) no neoadjuvant or adjuvant systemic therapy. Clinicopathological data, including age, sex, BMI, and tumor size, were recorded for each patient. All tumors were reviewed according to the 2016 World Health Organization (WHO) classification [18] and International Society of Urologic Pathologists (ISUP) grading [19]. Grades 1 and 2 were considered as low-grade disease, while grades 3 and 4 were considered as high-grade disease. Minimum four slides were reviewed for each case in order to minimize bias from intratumoral heterogeneity (ITH). Tumor grade was blindly reviewed by three independent pathologists, in order to minimize the inter-pathologist reliability. For a consensus diagnosis, at least 2 of the 3 pathologist’ diagnoses had to agree. Diameters of the primary tumors were obtained via CT imaging. Collected tumor tissues and plasma samples were stored in liquid nitrogen and – 80 °C until total RNA extraction. All PCR samples were analyzed at the same time. We assessed the expression levels of six genes (*PBRM1*, *BAP1*, *SETD2*, *KDM5C*, *FOXC2*, and *CLIP4*) reportedly associated with ccRCC, and four genes (*AQP1*, *DDX11*, *BAIAP2L1*, and *TMEM38B*) examined in our previous study involving RNA-seq analysis of aggressive ccRCC in clinical T1 stage [16, 17].

### Adiposity measurement

**Fig. 1:**
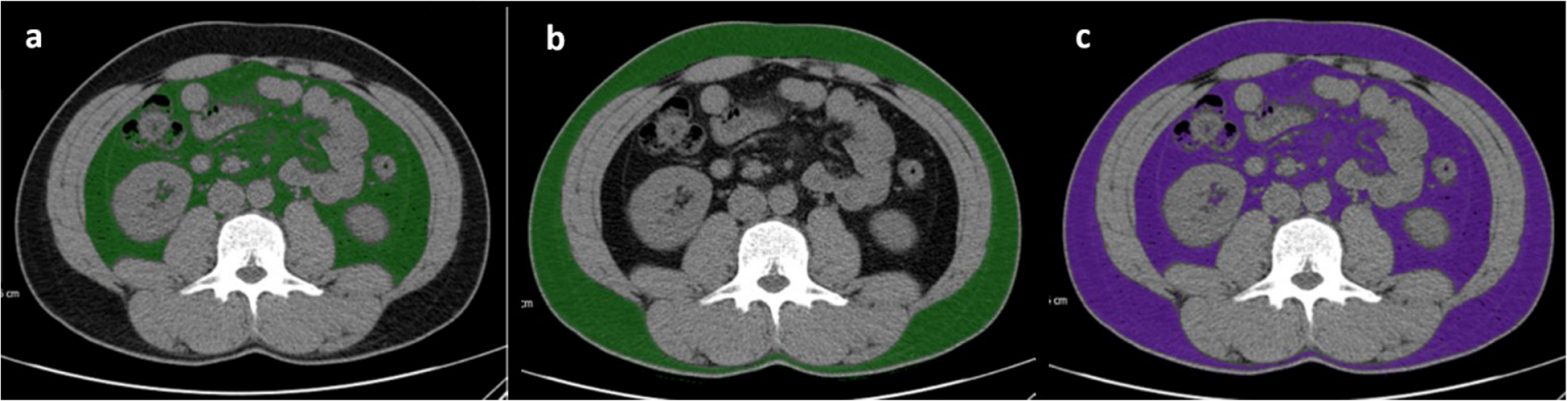
Computed tomography (CT) analysis of visceral and subcutaneous adipose tissue. Measurement of a visceral adipose tissue (VAT), b subcutaneous adipose tissue (SAT), and c total adipose tissue (TAT) by CT analysis software for a representative patient

As shown in Fig. 1, the contents of visceral adipose tissue (VAT), subcutaneous adipose tissue, and total adipose tissue were measured via preoperative CT of the mid-third lumbar vertebra region using Aquarius iNtuition Viewer, version 4.4.12 (TeraRecon, Foster City, CA, USA) with patients in a supine position. Preoperative CT was performed within 1 month before the surgery in order to minimize the change in body fat tissue over time. Previous studies have consistently used mid-third lumbar level for CT-defined image analysis [20]. Different body compositions were evaluated using predefined Hounsfield unit (HU) thresholds: − 190 to − 30 HU for subcutaneous adipose tissue, and − 150 to − 50 HU for VAT [20]. VAT% was calculated using the formula VAT% = [VAT/total adipose tissue] × 100 [20].

### Blood sample processing

Peripheral blood was collected from each participant, aliquoted into ethylenediaminetetraacetic acid (EDTA)-coated tubes, and centrifuged at 1600×*g* for 10 min at 4 °C. The plasma was then carefully transferred into new tubes and further centrifuged at 4000×*g* for 10 min at 4 °C and stored at – 80 °C until further analysis.

### RNA extraction and reverse transcription-quantitative polymerase chain reaction (RT-qPCR)

Total RNA was extracted from frozen tissues and plasma samples using TRIzol (Ambion, Life Technologies, USA). RNA isolated from 1 mL of plasma was dissolved in 20 μL of DEPC-treated water. The quantity and quality of RNA were assessed using a Nanodrop spectrophotometer (NanoDrop ND-1000, Thermo Scientific, Wilmington, DE, USA). Precisely 1 μg per sample was reverse-transcribed into first-strand cDNA using an iNtRon Maxime RT PreMix (Cat No. 25082; Intronbi, Seongnam, South Korea), following the manufacturer’s protocol. qPCR was performed using Power SYBR® Green Master Mix (Thermo Fisher, Cat No. A25742, USA) in a 10-μL reaction volume consisting of 5 μL of SYBR Green master PCR mix, 1 μL each of the forward and reverse primers (10 pmol), 1 μL of the diluted cDNA template, and UltraPure^TM^ distilled water (Invitrogen, NY, USA). The conditions for amplification are listed as follows: initial denaturation at 95 °C for 10 min; 40 cycles of denaturation at 95 °C for 15 s, annealing at 58 °C for 60 s, and elongation at 72 °C for 60 s; and final elongation at 72 °C for 5 min. qPCR was performed on an ABI StepOnePlus Real-Time PCR System (Applied Biosystems, Foster City, CA, USA). All measurements were conducted using *GAPDH* as the reference gene to normalize the relative expression levels of the target genes. PCR primer sequences are shown in Table S1. The relative gene expression was analyzed using the 2^–ΔΔCT^ method, and the results are expressed as percentage change compared to the control values. At least three replicates of RT-qPCR experiments were performed, and the results were analyzed by a blinded investigator.

### Statistical analyses

Data are presented as the means ± standard deviations or median (interquartile ranges) for continuous variables, and as percentage for categorical variables. Locally weighted scatterplot smoothing curves and Pearson’s correlation coefficient were used to assess the relationship between BMI and total adipose tissue, subcutaneous adipose tissue, VAT, or VAT%. High visceral adiposity was defined as a VAT level higher than the median VAT level. For univariate analysis, Wilcoxon test, Student’s *t* test, or one-way analysis of variance was used to compare continuous variables, and chi-square test or Fisher’s exact test was used to compare categorical variables. Multivariate logistic regression analyses were performed to identify predictors of high visceral adiposity and high ISUP grade, and odds ratios (ORs) with 95% confidence intervals (CIs) were calculated. SPSS software version 23.0 (IBM Corp., Armonk, NY, USA) was used for all statistical analyses. All statistical tests were two-tailed. *P* values less than 0.05 were considered statistically significant.

## Results

### Baseline demographics

The clinicopathological features of the study population (200 patients) are listed in Table 1. The median patient age was 52.0 years, and 76.5% of the patients were men. The median BMI was 24.8 kg/m^2^, and one (0.5%) patient was classified as underweight (less than 18.5 kg/m^2^), 101 (50.5%) as normal weight (18.5 to 24.9 kg/m^2^), 85 (42.5%) as overweight (25 to 29.9 kg/m^2^), and 13 (6.5%) as obese (30 kg/m^2^ or greater) according to the WHO BMI cutoff points [21]. The median tumor size was 2.0 cm, and 84 (42.0%) tumors were classified as high-grade.

**Table 1:** Clinicopathological characteristics of the study population according to gender

		Total (***n*** = 200)		Men (***n*** = 153; 76.5%)		Women (***n*** = 47; 23.5%)		***P***
Median age (range)		52.0 (43.0–60.8)		50.0 (42.0–54.0)		66.0 (60.0–68.0)		< 0.001
Median tumor size (cm) (range)		2.0 (1.4–2.9)		2.0 (1.4–2.8)		2.1 (1.4–3.6)		0.275
Median BMI (kg/m^2^) (range)		24.8 (23.5–27.7)		26.7 (24.2–28.6)		23.0 (21.9–24.2)		< 0.001
Median TAT (mm^2^) (range)		29,205 (24,937–37,647)		31,005 (26,140–39,174)		26,400 (20,232–27,316)		< 0.001
Median SAT (mm^2^) (range)		13,268 (10,127–17,695)		13,422 (10,127–18,016)		13,268 (11,218–17,042)		0.609
Median VAT (mm^2^) (range)		15,982 (11,302–19,879)		18,244 (14,495–20,584)		10,176 (4626–14,048)		< 0.001
Median VAT% (range)		51.4 (42.9–62.5)		55.0 (48.5–64.4)		40.7 (31.8–51.4)		< 0.001
No. comorbid conditions, *n* (%)								
Hypertension		72 (36.0%)		46 (30.1%)		26 (55.3%)		0.002
Hypercholesterolemia		16 (8.0%)		8 (5.2%)		8 (17.0%)		0.026
Diabetes		20 (10.0%)		11 (7.2%)		9 (19.1%)		0.025
No. smoking status, *n* (%)								
Never		75 (37.5%)		28 (18.3%)		47 (100.0%)		< 0.001
Former		70 (35.0%)		70 (45.8%)		0 (0.0%)		
Current		55 (27.5%)		55 (35.9%)		0 (0.0%)		
No. alcohol status, *n* (%)								
Never		69 (34.5%)		30 (19.6%)		39 (83.0%)		< 0.001
Former		40 (20.0%)		40 (26.1%)		0 (0.0%)		
Current		91 (45.5%)		83 (54.2%)		8 (17.0%)		
No. ISUP grade, *n* (%)								
Low-grade (1–2)		116 (58.0%)		105 (68.6%)		11 (23.4%)		< 0.001
High-grade (3–4)		84 (42.0%)		48 (31.4%)		36 (76.6%)		

Data are presented as medians (interquartile ranges) for continuous variables and as percentage for categorical variables. *P* values from the chi-square test or Fisher's exact test were used to calculate mean differences for categorical variables and those from Wilcoxon test were used to calculate mean differences for continuous variables. *BMI* body mass index, *SAT* subcutaneous adipose tissue, *TAT* total adipose tissue, *VAT* visceral adipose tissue, *VAT%* percentage of visceral adipose tissue

**Fig. 2:**
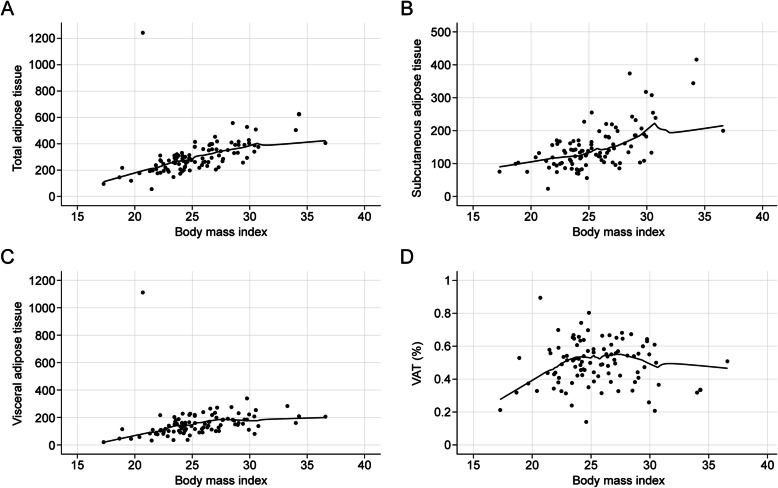
Correlation between body mass index (BMI) and adipose tissue parameters. Correlation between body mass index (BMI) and **a** total adipose tissue (TAT), **b** subcutaneous adipose tissue (SAT), **c** visceral adipose tissue (VAT), and **d** VAT%. Locally weighted scatterplot smoothing curves (blue line) were fitted in plots

The prevalence of hypertension, diabetes, and hypercholesterolemia in the patient population was 36.0%, 10.0%, and 8.0%, respectively (Table 1). As shown in Fig. 2, the total adipose tissue content [Pearson’s correlation coefficient (*r*) = 0.505, *P* < 0.001)] and the subcutaneous adipose tissue content (*r* = 0.536, *P* < 0.001) were significantly correlated with BMI. The VAT content (*r* = 0.274, *P* < 0.001) demonstrated significant correlations with BMI, however, VAT% (*P* = 0.207) did not. Age, hypertension, diabetes status, hypercholesterolemia, smoking status, and alcohol consumption differed significantly between men and women enrolled in the study (Table 1). Apart from subcutaneous adipose tissue, the adiposity variables, BMI, total adipose tissue, VAT, and VAT% were all significantly higher in men than in women.

### Clinicopathological characteristics according to VAT

Patients were stratified into quartiles (Q1 to Q4) according to the relative VAT contents (Table 2). High VAT content at diagnosis was associated with the male sex, an increased BMI, an increased prevalence of hypercholesterolemia, current smoking and alcohol status, as well as a low ISUP grade. Although significant differences were found among different VAT quartiles in terms of age, tumor size, prevalence of hypertension, they did not show any tendency.

**Table 2:** Clinicopathological characteristics according to visceral adipose tissue content

	Q1	Q2	Q3	Q4	***P***
Median age (range)	57.5 (51.0–62.0)	60.0 (46.0–68.0)	46.0 (39.0–57.3)	51.0 (42.0–54.0)	< 0.001
No. gender, *n* (%)					
Male	24 (48.0%)	33 (66.0%)	46 (92.0%)	50 (100.0%)	< 0.001
Female	26 (52.0%)	17 (34.0%)	4 (8.0%)	0 (0.0%)	
Median tumor size (cm) (range)	1.8 (1.2–2.8)	2.5 (1.8–3.6)	1.6 (1.4–1.7)	2.1 (1.2–2.9)	< 0.001
Median BMI (kg/m^2^) (range)	22.9 (21.9–24.6)	24.4 (23.0–26.6)	27.7 (24.1–29.0)	27.1 (25.1–29.2)	< 0.001
No. comorbid conditions, *n* (%)					
Hypertension	13 (26.0%)	26 (52.0%)	9 (18.0%)	18 (36.0%)	0.002
Hypercholesterolemia	0 (0.0%)	4 (8.0%)	4 (8.0%)	8 (16.0%)	0.034
Diabetes	8 (16.0%)	3 (6.0%)	6 (12.0%)	3 (6.0%)	0.261
No. smoking status, *n* (%)					< 0.001
Never	35 (70.0%)	22 (44.0%)	12 (24.0%)	6 (12.0%)	
Former	12 (24.0%)	22 (44.0%)	22 (44.0%)	14 (28.0%)	
Current	3 (6.0%)	6 (12.0%)	16 (32.0%)	30 (60.0%)	
No. alcohol status, *n* (%)					
Never	28 (56.0%)	20 (40.0%)	15 (30.0%)	6 (12.0%)	< 0.001
Former	7 (14.0%)	6 (12.0%)	9 (18.0%)	18 (36.0%)	
Current	15 (30.0%)	24 (48.0%)	26 (52.0%)	26 (52.0%)	
No. ISUP grade, *n* (%)					
Low-grade (1–2)	10 (20.0%)	20 (40.0%)	46 (92.0%)	40 (80.0%)	< 0.001
High-grade (3–4)	40 (80.0%)	30 (60.0%)	4 (8.0%)	10 (20.0%)	

Data are presented as medians (interquartile ranges) for continuous variables and as percentage for categorical variables. *P* values from one-way analysis of variance and chi-square test or Fisher’s exact test were used to determine mean differences for continuous and categorical variables, respectively, based on VAT quartiles. *BMI* body mass index, *SAT* subcutaneous adipose tissue, *TAT* total adipose tissue, *VAT* visceral adipose tissue, *VAT%* percentage of visceral adipose tissue

### Expression of target genes in frozen tissues and plasma according to high visceral adiposity

The relative mRNA levels of *DDX11* in the frozen tissue and plasma were significantly lower in patients with high visceral adiposity, revealed using univariate analysis (*P < 0.001* and *P = 0.016*, respectively) (Table 3). Multivariate logistic regression analysis of 10 target gene expressions from frozen tissue and plasma reported the significant association in frozen tissue *DDX11* expression according to high VAT (OR 0.676, 95% CI 0.587–0.779, *P* < 0.001) (Table 3). *FOXC2* and *AQP1* expression in frozen tissue were also significantly associated with high VAT in multivariate logistic regression analysis (*FOXC2*, OR 0.969, 95% CI 0.948–0.989, *P* = 0.003; *AQP1*, OR 0.410, 95% CI 0.212–0.793, *P* = 0.008) (Table 3).

**Table 3:** Expression of target genes in patients with clear-cell renal-cell carcinoma characterized by high/low visceral adiposity

	Low visceral adiposity	High visceral adiposity	***P***^***a***^	***P***^***b***^
mRNA expression (× 100)	(*N* = 100)	(*N* = 100)		
	*Frozen tissue*			
*FOXC2*	17.011 ± 33.556	7.896 ± 14.304	0.014	0.003
*CLIP4*	17.158 ± 32.455	9.365 ± 12.712	0.027	0.121
*PBRM1*	8.950 ± 11.257	7.032 ± 8.787	0.181	0.249
*SETD2*	1.476 ± 2.153	2.077 ± 2.316	0.059	0.759
*BAP1*	18.530 ± 59.910	1.649 ± 6.515	0.006	0.201
*KDM5C*	1.744 ± 2.704	1.954 ± 5.187	0.720	0.508
*AQP1*	1.283 ± 1.486	1.958 ± 0.981	< 0.001	0.008
*DDX11*	10.196 ± 10.477	1.890 ± 2.875	< 0.001	< 0.001
*BAIAP2L1*	3.007 ± 4.574	2.403 ± 3.139	0.277	0.051
*TMEM38B*	4.627 ± 7.711	7.055 ± 12.656	0.103	0.382
	*Plasma*			
*FOXC2*	16.708 ± 11.659	16.235 ± 8.215	0.740	0.705
*CLIP4*	16.848 ± 22.763	17.631 ± 22.080	0.805	0.895
*PBRM1*	2.452 ± 2.180	5.751 ± 41.896	0.417	0.099
*SETD2*	1.339 ± 1.690	2.346 ± 1.388	< 0.001	0.512
*BAP1*	98.325 ± 357.418	43.885 ± 244.658	0.210	0.633
*KDM5C*	13.468 ± 19.377	6.992 ± 9.286	0.003	0.776
*AQP1*	12.095 ± 19.380	8.017 ± 16.235	0.108	0.286
*DDX11*	5.484 ± 11.271	2.641 ± 2.657	0.016	0.081
*BAIAP2L1*	9.706 ± 15.835	4.965 ± 11.904	0.018	0.140
*TMEM38B*	17.087 ± 21.024	22.035 ± 33.570	0.213	0.523

Data are shown as the means ± standard deviations

^a^*P* values determined using Student’s t-test for univariate analysis

^b^*P* values determined using logistic regression for multivariate analysis

When we included the clinicopathological parameters and the mRNA expression of target genes that were significantly associated with VAT contents, the univariate logistic regression analysis revealed that gender, BMI, the prevalence of hypercholesterolemia, smoking and alcohol status, *DDX11* expression in frozen tissue, and ISUP score were significantly associated with high visceral adiposity (Table 4). Multivariate logistic regression analysis revealed that frozen tissue *DDX11* levels (OR 0.816, 95% CI 0.706–0.943, *P* = 0.006), and ISUP (OR 0.245, 95% CI 0.061–0.974, *P* = 0.046) were significantly associated with high visceral adiposity (Table 4).

**Table 4:** Predictors of high visceral adiposity in clinical T1a clear-cell renal-cell carcinoma

	Univariate		Multivariate	
	OR (95% CI)	***P***^***a***^	OR (95% CI)	***P***^***b***^
**Clinical parameters**				
Gender (reference male)	0.055 (0.019–0.162)	< 0.001	0.000 (0.000–0.000)	0.997
BMI (kg/m^2^)	1.496 (1.311–1.707)	< 0.001	1.148 (0.957–1.376)	0.138
Hypercholesterolemia (vs. none)	3.273 (1.018–10.523)	0.047	3.042 × e^10^	0.996
Smoking (vs. never)				
Former	3.353 (1.653–6.803)	0.001	0.284 (0.080–1.007)	0.051
Current	16.185 (6.650–39.392)	< 0.001	2.406 (0.606–9.545)	0.212
Alcohol (vs. never)				
Former	4.747 (2.055–10.964)	< 0.001	1.854 (0.383–8.965)	0.443
Current	3.048 (1.575–5.895)	0.001	0.374 (0.109–1.290)	0.119
**mRNA expression levels**				
*DDX11* (frozen tissue)	0.785 (0.726–0.850)	< 0.001	0.816 (0.706–0.943)	0.006
**ISUP nuclear grade** (reference low-grade)	0.070 (0.034–0.142)	< 0.001	0.245 (0.061–0.974)	0.046

^a^*P* values determined using logistic regression for univariate analysis

^b^*P* values determined using logistic regression for multivariate analysis. *BMI* body mass index

### Expression of target genes in frozen tissues and plasma according to high ISUP grade

The relative mRNA level of *DDX11* in the frozen tissue was significantly higher in patients with high ISUP grade, revealed using univariate analysis (*P < 0.001*) (Table 5). Multivariate logistic regression analysis of 10 target gene expressions from frozen tissue and plasma reported the significant association in frozen tissue *DDX11* expression according to high ISUP grade (OR 1.556, 95% CI 1.223–1.981, *P* < 0.001) (Table 5).

### Expression of DDX11 in frozen tissues according to high visceral adiposity and high ISUP grade in males and females

The separate analysis of males and females using logistic regression was performed due to the discrepancy of fat distribution between males and females. The results showed that DDX11 expression of frozen tissue for both significantly correlated with VAT (*P* < 0.001) and ISUP nuclear grade (*P* < 0.001) in males. Same results were also reported for females, showing that DDX11 expression of frozen tissue for both significantly correlated with VAT (*P* = 0.001) and ISUP nuclear grade (*P* = 0.004).

**Table 5:** Expression of target genes in patients with clear-cell renal-cell carcinoma characterized by high/low ISUP nuclear grade

	Low-grade	High-grade	***P***^***a***^	***P***^***b***^
mRNA expression (× 100)	(*N* = 116)	(*N* = 84)		
	*Frozen tissue*			
*FOXC2*	14.360 ± 29.842	9.820 ± 19.779	0.226	0.855
*CLIP4*	10.332 ± 15.012	17.307 ± 33.837	0.080	0.911
*PBRM1*	7.592 ± 8.283	8.543 ± 12.242	0.538	0.391
*SETD2*	2.615 ± 2.606	0.619 ± 0.633	< 0.001	0.095
*BAP1*	3.074 ± 12.171	19.778 ± 64.299	0.021	0.831
*KDM5C*	1.911 ± 4.806	1.763 ± 2.970	0.803	0.553
*AQP1*	2.337 ± 1.052	0.631 ± 0.903	< 0.001	0.117
*DDX11*	1.177 ± 2.103	12.763 ± 9.870	< 0.001	< 0.001
*BAIAP2L1*	3.155 ± 4.468	2.084 ± 2.931	0.042	0.448
*TMEM38B*	6.567 ± 12.125	4.838 ± 7.751	0.221	0.511
	*Plasma*			
*FOXC2*	16.649 ± 8.717	16.226 ± 11.721	0.770	0.490
*CLIP4*	16.241 ± 20.357	18.619 ± 24.948	0.460	0.333
*PBRM1*	5.672 ± 38.886	2.065 ± 1.880	0.397	0.538
*SETD2*	2.736 ± 1.488	0.610 ± 0.771	< 0.001	0.269
*BAP1*	26.289 ± 146.051	132.993 ± 435.106	0.033	0.520
*KDM5C*	6.804 ± 8.069	14.961 ± 21.136	0.001	0.964
*AQP1*	8.376 ± 16.698	12.376 ± 19.408	0.129	0.965
*DDX11*	3.545 ± 7.810	4.778 ± 8.911	0.311	0.953
*BAIAP2L1*	5.098 ± 12.002	10.425 ± 16.291	0.012	0.827
*TMEM38B*	21.343 ± 30.192	17.099 ± 24.749	0.277	0.950

Data are shown as the means ± standard deviations

^a^*P* values determined using Student’s *t* test for univariate analysis

^b^*P* values determined using logistic regression for multivariate analysis

## Discussion

In the present study, we identified the frozen tissue mRNA levels of *DDX11*, which is involved in cellular growth and division, as an independent prognostic factor for high visceral adiposity even after adjusting for clinicopathological parameters that are significantly associated with adiposity. To the best of our knowledge, this is the first study to investigate the association between the expression of genes potentially involved in ccRCC and adiposity, as well as to use visceral adiposity as a marker to infer the aggressiveness of small ccRCC.

BMI increases the relative risk for RCC [22]. However, the association between BMI and RCC prognosis remains controversial. Prior studies have mainly reported the proportional associations between BMI and RCC prognosis, whereas inverse, flat, or null associations have also been reported [23]. The inconsistencies among the results reported by these studies may be attributed to the use of BMI as a surrogate marker for obesity. Among various types of adipose tissue, VAT is the largest endocrine organ and produces hormones and cytokines that are related to carcinogenesis and tumor progression [15]. Subcutaneous adipose tissue and VAT share scant functional similarities other than their efficiency in energy storage [24]. VAT releases high levels of adipokines that are involved in inflammation and angiogenesis, including interleukin-6, vascular endothelial growth factor, and plasminogen activator inhibitor 1 [24]. In our study, BMI was not significantly correlated with VAT, indicating that the use of BMI would not provide consistent results in assessing the association between obesity and RCC prognosis. Among anthropometric measurements, the VAT content measured via CT has recently been examined for its utility in predicting the risk of cancer.

Five studies have examined the VAT content in patients with localized and/or advanced RCC [25]. Among these, three reported that low VAT contents are associated with poor prognosis in patients with RCC [25], and one study reported no association between VAT contents and overall mortality [26]. Park and colleagues reported that the lowest and the highest vs. the second quartiles of the VAT% are associated with a higher risk of recurrence [23]. Most studies have reported better prognosis in patients with high obesity, especially in those with localized SRMs [11, 23]. Moreover, Parker et al. reported that, in terms of aggressiveness, high BMI is associated with the presentation of a less aggressive form of RCC [9].

Among 28 studies that analyzed the body composition regarding the clinical outcomes of RCC in October 2016, 9 studies used fat index, which is the fat area divided by the height of the patients [25]. Since 19 studies used the fat area, we used it as well. However, there could be some bias in using only the fat area, not adjusted to the height of the patients.

Although subcutaneous adipose tissue is not associated with perioperative outcomes and survival in RCC, for other cancers, subcutaneous adipose tissue is reportedly associated with cancer-survival outcomes. Takamasa et al. reported that high subcutaneous adipose tissue volume in hepatocellular carcinoma is associated with better survival outcomes when treated with transcatheter intra-arterial therapies [27]. Moreover, leptin and adiponectin, which play a role in cancer biology, are both influenced by VAT and the subcutaneous adipose tissue [28, 29]. Therefore, although subcutaneous adipose tissue is not significantly associated with aggressive RCC, we should not overlook the unidentified importance of the subcutaneous adipose tissue.

*DDX11* expression, which is involved in cell-cycle progression, is used to predict tumor aggressiveness in clinically localized T1-stage ccRCC [16, 17]. Additionally, the inhibition of *DDX11* expression decreases the proliferation rate of melanoma cells and induces apoptosis [30]. In patients with lung adenocarcinoma, upregulated *DDX11* expression is associated with poor prognosis [31].

Consistent with the results of previous studies, our present study showed an inverse relationship between high visceral adiposity and the expression of *DDX11* mRNA in the frozen tissues. Previously, we showed that aggressive ccRCC, such as that associated with synchronous metastasis, recurrence, and/or cancer-specific death, is also associated with the upregulated expression of *DDX11* mRNA in both plasma and frozen tissues. Most studies showed that high VAT contents are associated with improved prognosis; thus, we suggest that less aggressive ccRCC is likely associated with high VAT contents. The results obtained in our present study indicated that non-aggressive ccRCC is associated with high VAT contents and low expression of *DDX11*. Based on these results, conservative therapeutic options, such as ablation and active surveillance, would be prudent strategies for treatment of patients with small ccRCC, a high VAT, and decreased *DDX11* expression.

The underlying mechanism that links visceral adiposity to the upregulation of *DDX11* expression is unknown. The pathogenesis of how *DDX11* is involved in adipogenesis and cancer progression is undetermined although some suggestions for the plausible mechanisms might be possible. *DDX11*, a DNA-dependent ATPase and helicase, plays an important role in the cohesion of chromosome arms and centromeres [32]. The depletion of *DDX11* results in mitotic failure because the replicated chromosomes fail to segregate after prometaphase [32]. *DDX11* expression may be associated with the G1–S phase of the cell-cycle and the pathways involved in DNA replication [32]. Recent studies suggest that adipocyte differentiation, lipogenesis, and lipolysis are strongly modulated by cell-cycle regulators, which control the checkpoints for cell duplication [33]. Our results suggest that *DDX11*, which is involved in cell-cycle regulation, may be associated with VAT generation. Because the exact pathways connecting cell-cycle regulation and adiposity remain unknown, future studies should elucidate the mechanisms underlying visceral adiposity and *DDX11* expression. We are under in vitro experiments on how *DDX11* affects both adipogenesis and cancer progression. This study suggested the possible mechanisms linking VAT, *DDX11*, and tumor aggressiveness and provided support for future studies including in vitro experiments we are undergoing.

Although *FOXC2* and *AQP1* were not significantly associated with ISUP nuclear grades, they were significantly associated with VAT. *FOXC2* and *AQP1* were the first to be identified as the significant genes associated with ccRCC aggressiveness such as synchronous metastasis and ccRCC-specific death in on our previous studies [16, 17, 34]. Our group was the first to identify *FOXC2* as a biomarker of aggressive ccRCC [16, 34]. One study reported upregulation of *FOXC2* in aggressive ccRCC [34], while downregulation of *FOXC2* in aggressive ccRCC was observed in the subsequent study [16]. We believe that these differences might have resulted from different definition of aggressiveness. Ahn et al. analyzed the biomarkers that are associated with synchronous metastasis [34], while Park et al. demonstrated the biomarkers associated with a tumor exhibiting synchronous metastasis, recurrence, or cancer-specific death, and synchronous metastasis [16]. According to other studies, the results are controversial as well, with one study reporting upregulation of *FOXC2* in association with cancer metastasis and epithelial-mesenchymal transition [35, 36], while other study reports *FOXC2* upregulation acts as a checkpoint to inhibit epithelial cell dedifferentiation [37]. Although several studies have reported the usefulness of urine AQP1, few studies reported analysis of the frozen tissue AQP1 in RCC. Huang et al. demonstrated that frozen tissue *AQP1* expression was significantly differed according to RCC subtype-specific expression, and its expression level provided prognostic information for ccRCC patients [38].

Our study was the first to evaluate the association between visceral adiposity and mRNA expression of target genes. Moreover, our results enabled us to easily infer the aggressiveness of ccRCCs using visceral adiposity calculated from preoperative CT without any invasive diagnostic modalities. Although mutations in each of these kidney cancer genes result in dysregulation of metabolic pathways, suggesting that kidney cancer is a disease of cell metabolism [39], no studies have attempted to find the association of gene expression or tumor aggressiveness of ccRCC with metabolic factors such as adiposity. We believe that this study could be the cornerstone for the ccRCC metabolism in association with biomarker expression and tumor aggressiveness.

Our study has a few limitations. First, the ITH of primary tumors is a considerable problem, even in SRMs [25]. ITH causes sampling bias in conventional needle biopsies. Clinical trials are currently examining the use of circulating tumor DNA in plasma to overcome the limitations imposed by ITH. Second, we could not use visceral adiposity and target-gene expression to predict prognostic indexes, such as cancer-specific or progression-free survival, in patients with ccRCC, owing to the short follow-up period. Only one patient developed recurrence among the 200 patients included in this study, and no other patients developed recurrence, metastasis, or cancer-specific death. WHO/ISUP grading system has several advantages over the former Fuhrman grading system, that are easier to apply, more reproducible and clinically relevant with its relevance for prognosis and serving as an alternative means of categorizing tumors for future patient management [19, 40]. Since high nuclear grade is currently the most important and significant prognostic factor for predicting oncological outcomes [41], we evaluated the association between the expression of biomarkers and a high nuclear grade. Third, the study population was relatively small. Future studies should investigate the correlations among visceral adiposity, target-gene expression, and prognosis in large populations of patients with ccRCC.

## Conclusions

In the present study, we investigated the association between visceral adiposity and target-gene mRNA expression in patients with localized small ccRCC. *DDX11* mRNA levels in the frozen tissues and plasma are significantly associated with high visceral adiposity. Quantifying the VAT contents in preoperative CT scans will enable us to infer the aggressiveness of small ccRCCs in clinical practice. The role of *DDX11* in the regulation of VAT warrants further investigation in future studies.

## Supplementary Information

**Additional file 1: Table S1**

## Data Availability

Data are not publically available to other researchers and only be permitted after the approval of IRB.

## Abbreviations

CI: Confidence intervals
CT: Computed tomography
HU: Hounsfield unit
ISUP: International Society of Urologic Pathologists
ITH: Intratumoral heterogeneity
KHIDI: Korea Health Industry Development Institute
OR: Odds ratios
RCC: Renal-cell carcinoma
SAT: Subcutaneous adipose tissue
SRM: Small renal masses
TAT: Total adipose tissue
VAT: Visceral adipose tissue
WHO: World Health Organization
