# Supplementary material for: Association between visceral adiposity and DDX11 as a predictor of aggressiveness of small clear-cell renal-cell carcinoma: a prospective clinical trial
Source: Cancer Metab. 2021 Apr 6;9:15. doi: 10.1186/s40170-021-00251-y (PMC8025550; doi:10.1186/s40170-021-00251-y)
Supplement: Supplementary file 1 — Additional file 1: Table S1 [file 40170_2021_251_MOESM1_ESM.docx]

**SUPPLEMENTARY MATERIALS**

**Table S1. PCR primer sequences**

|  | **Primer sequences** | |
| --- | --- | --- |
|  | **Sense** | **Antisense** |
| **FOXC2** | 5′-GAT CAC CTT GAA CGG CAT CT-3′ | 5′-ACC TTG ACG AAG CAC TCG TT-3′ |
| **CLIP4** | 5′-GCA TCA TGC CAG GAA ATT CT-3′ | 5′-TTT GTT GGA CCT GAG GAA CC-3′ |
| **PBRM1** | 5′-TGA TGG CCA ACA AGT ACC AA-3′ | 5′-AGA TCA AAG ACT CCG GCT CA-3′ |
| **SETD2** | 5′-TCA CAA GGC AGA CTC AGT GG -3′ | 5′-CTG CTG TCT TGG GCT TTT TC-3′ |
| **BAP1** | 5′-GCC TGA GGA GTC CAA GTC AG-3′ | 5′-CTG GAG GCT TCA CCA CTA GC-3′ |
| **KDM5C** | 5′-GTC ATT TGC AAC CCC TGA GT-3′ | 5′-AAT GGG ATG AGG GGT AAA GG-3′ |
| **AQP1** | 5′-CAA CTT CAG CAA CCA CTG GA-3′ | 5′-GTC GGC ATC CAG GTC ATA CT-3′ |
| **DDX11** | 5′-TCT CTT GGC TCC GTG ACT TT-3′ | 5′-TTT AGT CGG TCC ACC AGG TC-3′ |
| **BAIAP2L1** | 5′-GGC AGG AGA CCT GTG TTG AT-3′ | 5′-AGC CTG AGG AGT TCC AGA CA-3′ |
| **TMEM38B** | 5′-TCG GGA ATG AAG GAA GTG AC-3 | 5′-AGC CAT TCA TCA CCT TCT GG-3′ |
| **GAPDH** | 5′-CAG CCT CAA GAT CAT CAG CA-3′ | 5′-GGT GCT AAG CAG TTG GTG GT-3′ |
|  | | |
